# Unveiling the Unknown: A Case Report of Undifferentiated Pleomorphic Sarcoma in the Heart

**DOI:** 10.1177/11795468251408695

**Published:** 2026-09-07

**Authors:** Dina Fa Alwaheidi, Mohd Lateef Wani, Ashfaq Patel

**Affiliations:** 1 Cardiac Surgery Department, Hamad Medical Corporation, Doha, Qatar; 2 Cardiology Department, Hamad Medical Corporation, Doha, Qatar

**Keywords:** sarcoma, cardiac masses, heart failure

## Abstract

Undifferentiated pleomorphic sarcoma (UPS) of the heart is an exceedingly rare and highly aggressive tumor. We present the case of a 66-year-old male with a history of stage I prostatic acinar adenocarcinoma who was incidentally found to have a left atrial mass during cancer workup. Multimodal imaging (echocardiography, CMR, PET-CT) initially suggested a benign lesion. The patient underwent surgical excision of the mass, which revealed UPS confirmed by histopathology and immunohistochemistry. Postoperatively, the patient experienced right-sided heart failure, recurrent skin ulcers requiring grafting, and atrial arrhythmia. Despite complications, he stabilized and started chemotherapy and oncologic follow-up. This case highlights the diagnostic pitfalls of cardiac UPS, the necessity for tissue confirmation, and the role of coordinated multidisciplinary care.

## Introduction

Primary cardiac sarcomas are exceedingly rare, accounting for less than 0.1% of all malignant tumors, with undifferentiated pleomorphic sarcoma (UPS) being an uncommon histologic subtype.^1^ Due to their rarity and nonspecific presentation, these tumors often mimic benign cardiac masses such as myxomas, delaying diagnosis and impacting outcomes. Reporting such cases is crucial to raise awareness of diagnostic pitfalls and highlight evolving imaging and surgical strategies. This case illustrates an unusual presentation of left atrial UPS with misleading imaging features and underscores the importance of integrating multimodal imaging with histopathological correlation

## Case Presentation

A 66-year-old male presented with lower urinary tract symptoms, including increased urinary frequency and nocturia. Subsequent urologic evaluation and prostate biopsy revealed stage I prostatic acinar adenocarcinoma. While awaiting definitive urologic treatment, a routine preoperative workup—including transthoracic echocardiography—incidentally detected a left atrial mass protruding into the mitral valve orifice, without associated valvular dysfunction. Remarkably, the patient denied any cardiac symptoms such as chest pain, dyspnea, or palpitations at the time of discovery. Following this initial assessment, the patient underwent a robotic-assisted prostatectomy, which was performed in a medical facility located abroad, and subsequently, he was referred for a more detailed evaluation concerning the mass found in his left atrium. Summary of Laboratory and echo Investigations included in Table 1.

**Table 1.** Serial Laboratory and Cardiac Function Tests Revealed Evolving Biventricular Dysfunction and Elevated Cardiac Biomarkers During Both Postoperative and Oncologic Follow-Up.

Parameter	Reference range	Preoperative	Postoperative (POD 3)	At readmission (POD 21)	October 2025 (latest follow-up)
Hemoglobin (g/dL)	13.5-17.5	12.8	10.2	8.7	13.7
White blood cell count (×10^9^/L)	4.0-10.0	7.6	11.2	9.4	8.3
Platelet count (×10^9^/L)	150-450	220	280	195	305
Creatinine (µmol/L)	60-110	95	108	115	103
BUN (mmol/L)	2.5-7.1	6.2	8.4	9.1	7.0
NT-proBNP (pg/mL)	<125	—	1450	2300	350
Troponin I (ng/L)	<34	—	56	28	<10
CRP (mg/L)	<5	7.1	48.0	35.5	<5
PSA (ng/mL)	<4.0	5.8	—	—	0.01
LDH (U/L)	125-220	—	285	310	210
Ki-67 index (%)	—	—	40% (tumor biopsy)	—	
RVSP (mmHg, from ECHO)	—	65.7	55.2	46.24	48.4
LVEF (%)	>55%	59.5	47	36	55
GLS (%)	–18% to –22%	–14.2	–11.1	–10.2	10.2

**Figure 1.**
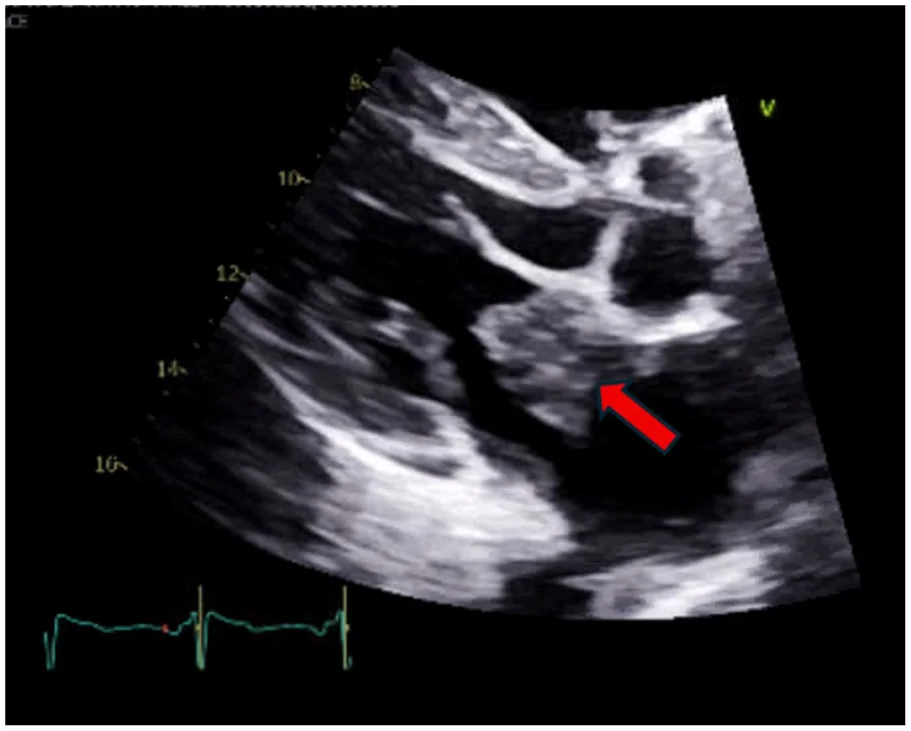
Preoperative transthoracic echocardiogram. Echo images showing a definite medium-size (2.7 cm × 2.7 cm), broad based, spherical, mobile tumor in the atrial cavity (red arrow), attached to the inter-atrial septum very near to the base of the anterior mitral leaflet.

Echo revealed a mass characterized as broad-based and spherical in shape, which exhibited mobility within the atrial cavity and was firmly attached to the interatrial septum as shown in (Figure 1), situated very close to the base of the anterior mitral leaflet, raising concerns regarding its potential impact on cardiac function. This TTE image raised initial suspicion for a benign lesion, most likely a myxoma, due to its morphology.

Further evaluation was conducted utilizing CMR showing it to be dilated and containing an oblong, broadly lobulated lesion within the atrial cavity that demonstrated mild lobulation; this lesion was also affixed to the interatrial septum in proximity to the mitral valve leaflet orifice, measuring approximately 4.6 by 2 cm.

**Figure 2.**
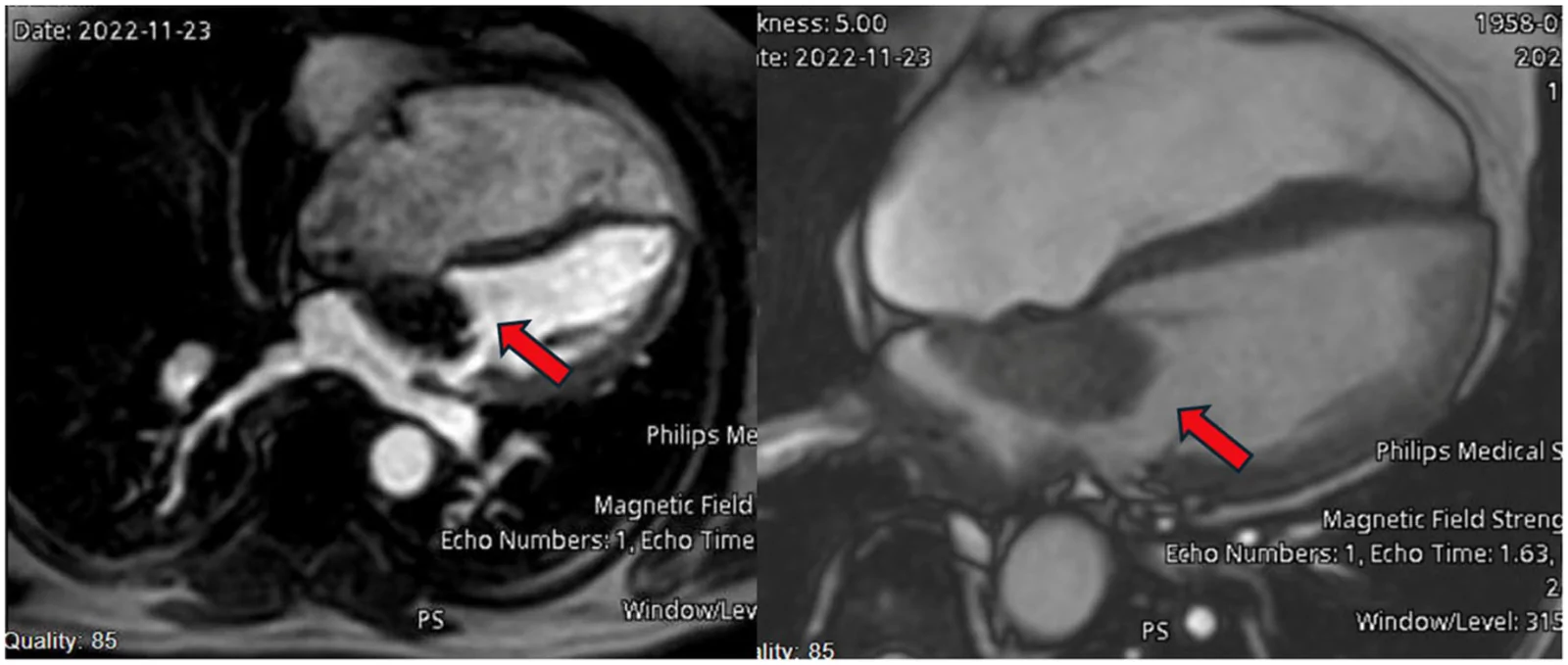
MRI images T1,T2-weighted sequences. Showing dilated left atrium with an oblong to broad-based lobulated lesion (red arrow) within the left atrial cavity with mild lobulation and attached to the interatrial septum near the base and abutting the mitral valve leaflet orifice, it measures around 4.6 cm × 2 cm in its maximum dimension. The mass has isointense signal intensity on the T1-weighted sequences, iso to mildly hyperintense on the T2-weighted sequence, the perfusion images shows no vascularity.

**Figure 3.**
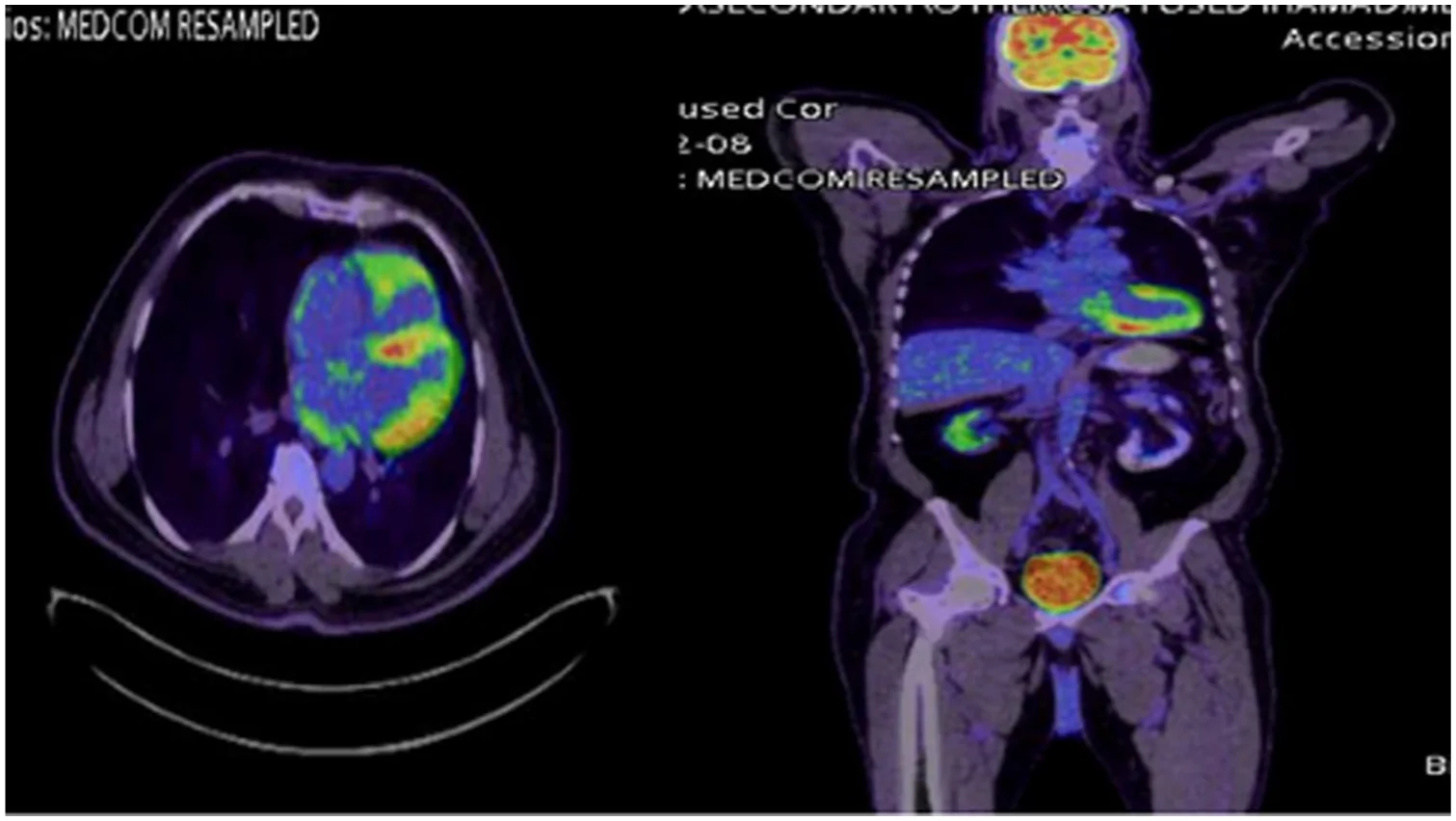
Fused PET/CT images of the thorax and whole body. Fused axial (left) and coronal (right) PET/CT images demonstrating a hypermetabolic left atrial mass with high FDG uptake. No evidence of distant metastases was observed on whole-body screening.

The mass exhibited an isointense signal intensity on T1-weighted imaging, while it appeared iso to mildly hyperintense on T2-weighted sequences; interestingly, the perfusion images indicated a lack of vascularity, and early imaging did not reveal any definitive enhancement of the lesion, although delayed sequences demonstrated strong, homogeneous hyperenhancement with a few areas exhibiting signal voids. The features observed in the CMR imaging (Figure 2) were quite suggestive of a fibrous tissue mass lesion, likely indicative of a fibroma; however, other differential diagnoses such as fibrosarcoma and hemangioma were also considered, prompting further investigation through a PET-CT scan, which ultimately showed no increased (FDG) uptake within the left atrial mass as shown in Figure 3

Despite recommendations for surgical intervention, the patient postponed the procedure due to personal issues, expressing a continued insistence on his asymptomatic status, which led him to further defer his operation. Meanwhile, follow-up echocardiographic imaging revealed a concerning increase in the size of the mass, accompanied by a progression of right ventricular (RV) dilation and a decrease in its functional capacity, with the right ventricular systolic pressure (RVSP) measuring approximately 65 mmHg, along with a gradual decline in his hemoglobin levels. Eventually, the patient presented with shortness of breath and was admitted to the hospital with an acute pulmonary edema. patient was consented for surgical intervention.

**Figure 4.**
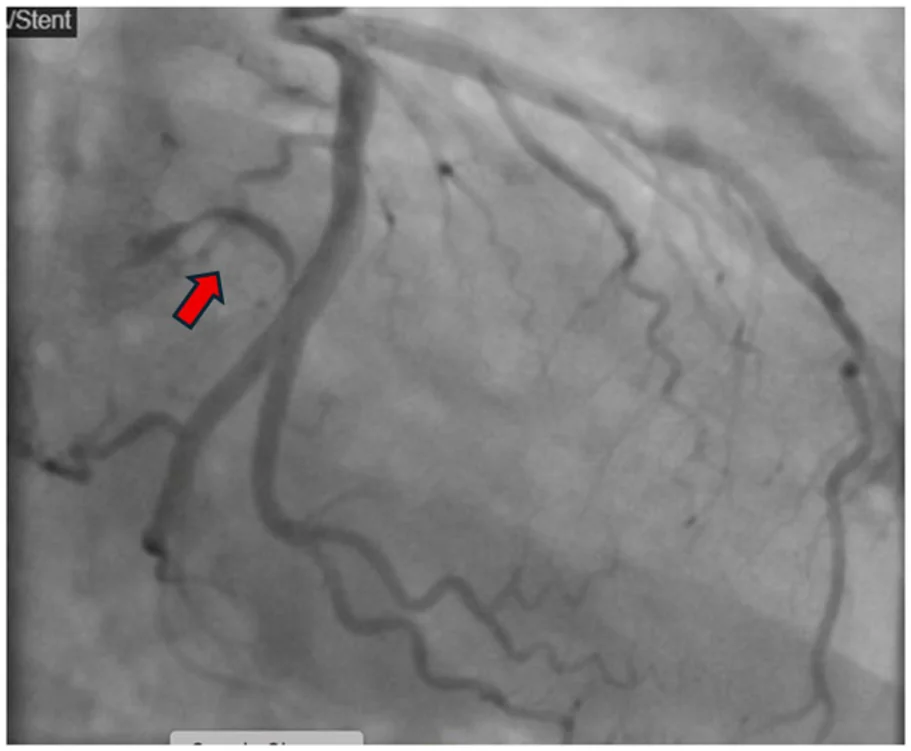
Coronary angiogram demonstrating tumor vascular supply. Selective coronary angiogram revealing a branch from the circumflex artery supplying the hypervascular mass located in the posterior left atrium (red arrow).

Coronary angiogram showed a branch from the circumflex artery supplying the mass located in the left atrium Figure 4. A repeat PET demonstrated minimal uptake in the left atrial mass, suggesting the possibility of a myxoma or fibroma, while high-grade malignancy was deemed less likely due to the observed low-level uptake, in addition to detecting an FDG-avid nodule in the left thyroid gland.

Surgical excision of the left atrial mass through a median sternotomy approach, Full bypass then right atrial incision followed by septal incision proximal to the fossa ovalis, facilitating access to the mass.

**Figure 5.**
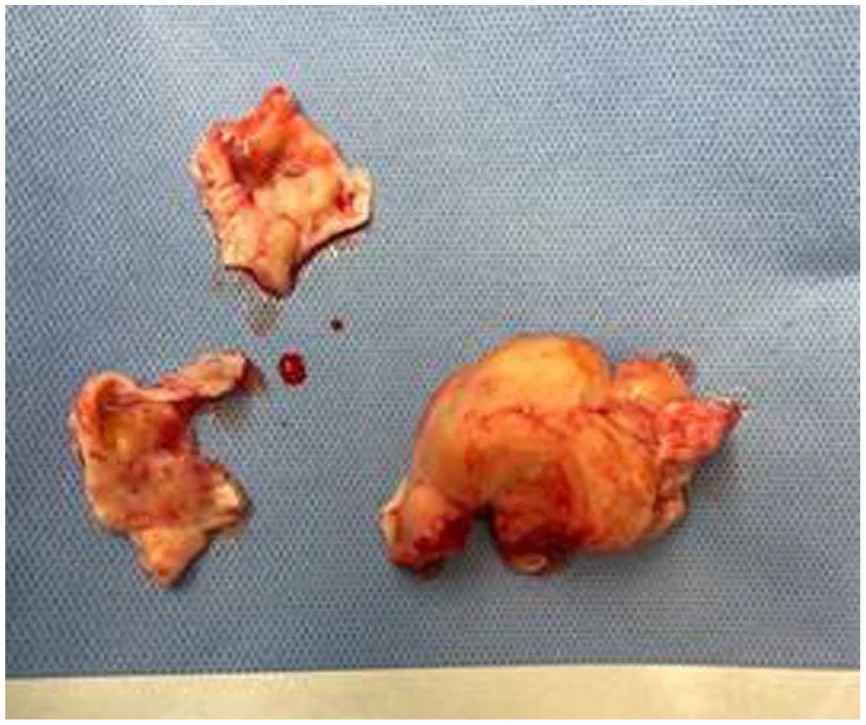
Gross tumor specimen following surgical resection. Figure shows gross surgical specimen of the undifferentiated pleomorphic sarcoma (UPS) involving the left atrium, demonstrating a large, lobulated, tan-pink mass with heterogeneous consistency and multiple nodular extensions. The tumor was resected en bloc in several fragments, the largest of which displays a glistening, myxoid surface. This appearance mimicked benign myxoma macroscopically, which is a common diagnostic pitfall in cardiac UPS.

The mass was carefully debulked in its entirety along its attachment points, which included a portion of the interatrial septum as well as the roof of the left atrium. The mass was noted to be protruding into the mitral valve, and moreover, a congenital cleft was identified between the A2 and A3 segments of the mitral valve, which was subsequently repaired using a 5 to 0 proline suture for reinforcement. Roof of the left atrium was primarily closed following the excision, and the septum was reconstructed using an autologous pericardial patch, ensuring structural integrity and proper function of the cardiac anatomy postoperatively. R0 resection was achieved, with close but negative margins (<2 mm). Intraoperative inspection and TEE confirmed no gross infiltration of adjacent valvular or annular tissue and no remenant mass was evident. The excised mass appeared well-encapsulated. Gross appearance remained consistent with myxoma, further misleading the clinical team until final pathology confirmed UPS as shown in Figure 5.

The immediate intra-operative TEE showed Good LV function with no residual tumor. Postoperative recovery was marked by complications including atrial flutter accompanied by a rapid ventricular response; which was managed with amiodarone in conjunction with beta-blockers to stabilize the patient’s cardiac rhythms. Patient had superficial skin ulcerations, which exhibited central necrosis in both of his heels, raising concerns regarding the underlying etiology. Histopathological analysis suggested that the observed ulcerations were primarily related to stasis rather than indicative of pyoderma gangrenosum.

Patient was readmitted due to the exacerbation of decompensated right-sided heart failure. Echocardiogram revealed moderately reduced left ventricular systolic function, the presence of a severely dilated right ventricle, and significantly impaired right ventricular function, the calculated area of the right atrium was found to be approximately 28.2 cm^2^, with concomitant moderate tricuspid regurgitation and an elevated RVSP measured at 46.24 mmHg, indicating a substantial burden on the right side of the heart. Right heart cath was performed as shown in Table 2. No significant pulmonary hypertension or elevated resistance was noted, the mild elevation in RVSP noted by echocardiography was likely due to the mass pressure. Despite the improvement in his cardiac condition, the ulcers present on both of his heels showed no signs of healing, prompting the decision to proceed with a surgical intervention involving skin grafting harvested from the donor area of his left thigh. In the interim, the patient’s cardiac status remained stable, and he did not experience any further exacerbations related to heart failure. The clinical course is summarized in Table 3, which outlines key milestones including the initial cardiac imaging, surgical resection, readmissions, and commencement of systemic therapy.

**Table 2.** Right Heart Cath Result.

Parameter	Value
Right atrial pressure (RA)	12 mmHg
Right ventricular pressure (RV)	35/5/4 mmHg
Pulmonary artery pressure (PA)	32/20/25 mmHg
Pulmonary capillary wedge pressure (PCWP)	12/13/12 mmHg
Cardiac output (fick method)	6.44 L/min
Cardiac index (fick)	3.44 L/min/m^2^
Cardiac output (thermodilution)	6.30 L/min
Cardiac index (thermodilution)	3.44 L/min/m^2^
Systemic vascular resistance (SVR)	15.0 Wood units
Pulmonary vascular resistance (PVR)	2.0 Wood units
Cardiac power output	1.4 W/m^2^
Pulmonary artery pulsatility index (PAPI)	1.70
Interpretation	Normal biventricular filling pressures and preserved cardiac output

**Table 3.** A Comprehensive Timeline of the Patient’s Clinical Course, Including Key Surgical and Oncologic Milestones.

Date	Event/finding	Details/interpretation
10/03/2024	Surgical resection	Left atrial mass excision, mitral valve cleft repair
11/03/2024	Post-op POD 1	Transferred to HDU
13/03/2024	POD 3	Atrial flutter noted, started anti-coagulation
18/03/2024	POD 8	Echo: mild LV dysfunction, severely dilated RV, residual LA mass 3.2 × 1.7 cm
21/03/2024	POD 11	TEE: vascularized LA roof mass; tumor versus hematoma suspected
25/03/2024	Pathology result-prelimineray	Disrupted atrial myxoma.
31/03/2024	POD 21	Histopathology: undifferentiated sarcoma
27/05/2024	1sr readmission	Heart failure with persistent atrial flutter; new thrombus/mass in LA roof
30/05/2024	MRI	Mass regressed; thrombus suspected; moderate RV dysfunction
03/07/2024	Non-healing wounds at both heels	Debridement; 10 d later followed by skin grafting
15/10/2024	Follow-up MRI	Mild progression of LA roof lesion, no new myocardial fibrosis
21/10/2024	MDT discussion	Serial MRI follow-up planned
27/10/2024	TEE	Mass still present, partial contrast filling suggests neoplasm
25/12/2024	Pathology updated	Undifferentiated pleomorphic sarcoma
31/12/2024	2nd readmission	Cardioversion to NSR, TEE showed no thrombus
Sep-Aug 2025	MDT\cardio-oncology FU	Recurrence of atrial flutter; CCF; palliative chemo started
28/08/2025	FDG PET	Persistent moderate uptake in LA mass; no distant metastasis

## Histopathological Evaluation

Initial histopathological assessment following surgical excision of the left atrial mass on March 25, 2024, suggested a disrupted atrial myxoma, with no evidence of malignancy and clear inked margins. However, due to atypical features and clinical suspicion, the slides were referred for an external pathology consult. A detailed second opinion from the Mayo Clinic, finalized on Dec 2024, revised the diagnosis to undifferentiated pleomorphic sarcoma (UPS) of the heart. Microscopic evaluation revealed spindled neoplastic cells with moderate pleomorphism in a variably myxoid background, lacking features of myxoma. Immunohistochemical staining demonstrated reactivity for CD34, ERG, and weak podoplanin, while markers such as calretinin, CD31, AE1/AE3, SMA, and S100 were negative, consistent with a diagnosis of UPS. The Ki-67 index was low (<5%), and MDM2 showed nuclear positivity, supporting the malignant diagnosis. Notably, no definitive myxomatous background or perivascular ring structures were identified, effectively ruling out myxoma.

The patient was urgently discussed in a cardio oncology MDT meeting and referred to medical oncolog. He completed 2 cycles of gemcitabine and docetaxel (gemcitabine 900 mg/m^2^ on Days 1 and 8, and docetaxel 100 mg/m^2^ on Day 8), which were well tolerated. A follow-up PET-CT in August 2025 revealed persistent moderate FDG uptake within the left atrial mass with mild-to-moderate reduction in avidity compared to baseline. Importantly, there was no evidence of nodal or distant metastatic disease, indicating stable disease under treatment. Then, he received dacarbazine-based chemotherapy. Evaluation in Heart Failure Clinic noted continued symptoms (NYHA Class II), persistent arrhythmias, and laboratory evidence of decompensation (NT-proBNP 4358). Echocardiography reaffirmed worsening RV function and increasing pulmonary pressures (PVR 3.3 WU).

The patient’s clinical status is now consistent with chronic right heart failure and atrial arrhythmias, likely related to progressive tumor burden. He is currently receiving palliative chemotherapy and has been advised to seek further oncologic evaluation abroad. Cardiac-wise, management is focused on rate control and symptom relief (Table 2).

## Discussion

Undifferentiated pleomorphic sarcoma (UPS), formerly classified under malignant fibrous histiocytoma, is a rare and aggressive mesenchymal tumor that infrequently affects the heart. It accounts for a minority of primary cardiac sarcomas, which themselves represent only 25% of all primary cardiac tumors—of which benign myxomas dominate the incidence spectrum. Among cardiac sarcomas, the left atrium remains the most common site, followed by the right atrium and other chambers.^1-3^

The clinical presentation of cardiac UPS is highly variable and often insidious, typically manifesting as dyspnea, chest discomfort, constitutional symptoms, or embolic phenomena. However, its signs frequently mimic those of benign entities such as myxomas, which poses a significant diagnostic challenge.^4^ Imaging remains the cornerstone for preoperative characterization. On echocardiography and cardiac MRI, UPS may appear as a broad-based, lobulated, or polypoid lesion, with features such as irregular borders, heterogeneous enhancement, and infiltration of surrounding structures. These features contrast with myxomas, which are generally well-circumscribed, pedunculated, and arise from the fossa ovalis of the interatrial septum.^5-7^ PET imaging may further aid in distinguishing benign from malignant lesions by evaluating metabolic activity; nonetheless, as in our case, low FDG uptake does not rule out malignancy.^8^

UPS typically presents as large, irregular masses that may exhibit central necrosis, making them easily mistaken for myxomas. Definitive diagnosis is usually achieved through histopathological examination following surgical resection, revealing undifferentiated cells with pleomorphic characteristics. The potential for misdiagnosis underscores the importance of considering UPS in the differential diagnosis of cardiac masses.

Histopathological evaluation remains the definitive diagnostic modality. UPS demonstrates highly pleomorphic spindle cells, numerous mitoses, areas of necrosis, and a high Ki-67 index.^9-11^

Surgical resection with negative margins is the cornerstone of management and offers the best chance at local control. However, complete excision is often impeded by the proximity of the tumor to vital intracardiac structures. Recurrence after incomplete resection is common, with some studies reporting recurrence rates as high as 70% to 80%.^12^ In the retrospective study of the.

The role of adjuvant chemotherapy and radiotherapy remains controversial. Regimens such as doxorubicin-ifosfamide or gemcitabine-docetaxel have been used in various extrapulmonary soft-tissue sarcomas and applied in cardiac UPS with variable success. Our patient received gemcitabine-docetaxel and demonstrated stable disease radiographically, without evidence of nodal or distant metastasis on serial PET imaging. Radiotherapy has limited use due to concerns of cardiotoxicity and uncertain efficacy .Immunohistochemistry typically shows positivity for vimentin and negativity for lineage-specific markers such as cytokeratin, desmin, S100, and CD34, thereby excluding epithelial, myogenic, neural, and vascular differentiation respectively.^13,14^

French Sarcoma Group showed that only surgical resection is associated with a perspective of prolonged survival. Chemotherapy is associated with a better outcome in non-resected patients. In our case, the tumor’s extension to the roof of the left atrium and anterior mitral leaflet necessitated a complex resection and reconstruction.^15^

Recent advances in molecular profiling of soft tissue sarcomas have opened avenues for targeted therapies. Studies have identified potential targets such as PDGFR, MDM2 amplification, or complex gene fusions that may respond to tyrosine kinase inhibitors or other novel agents. Although such approaches remain investigational in cardiac sarcomas, their application could be transformative for patients with unresectable or metastatic disease.^16,17^ Furthermore, whole-genome or RNA sequencing may help in better subclassification of UPS, which is currently a diagnosis of exclusion.^18^

Prognosis remains guarded. Median overall survival ranges from 6 to 18 months depending on the completeness of resection and presence of metastases. In a pooled analysis of cardiac UPS cases, survival beyond 24 months was rare without aggressive surgical management and close oncologic follow-up. Our case underscores the importance of maintaining high clinical suspicion, early referral to a multidisciplinary team, and long-term surveillance.^19^

Finally, a key learning point in this case relates to imaging differentiation. Cardiac MRI may provide clues—UPS often appears as a larger, infiltrative, irregular mass with heterogeneous signal and enhancement, unlike myxomas which are typically smooth, mobile, and show uniform enhancement. PET-CT may aid in differentiation by revealing hypermetabolic activity in malignant tumors, although false negatives can occur. A combined imaging approach with tissue diagnosis remains vital in such ambiguous cases. Although Definitive diagnosis is usually achieved through histopathological examination revealing undifferentiated cells with pleomorphic characteristics which can also be subjected to error.^20^ The potential for misdiagnosis underscores the importance of considering UPS in the differential diagnosis of cardiac masses.

The major challenges in evaluating cardiac masses is differentiating malignant tumors such as undifferentiated pleomorphic sarcoma (UPS) from benign lesions like myxomas, which are far more prevalent. While both entities can present as left atrial masses and may appear similar on echocardiography, certain imaging characteristics can aid in distinguishing between them. Cardiac myxomas typically arise from the interatrial septum, especially near the fossa ovalis, and display a smooth surface, homogeneous echogenicity, and well-demarcated borders with occasional stalk attachment. In contrast, sarcomas often present as broad-based, heterogeneous, and lobulated masses with infiltrative borders, sometimes extending beyond the atrial cavity or invading adjacent cardiac structures. Cardiac MRI further enhances diagnostic precision, as myxomas tend to show isointense or mildly hyperintense signals on T2-weighted imaging with minimal contrast uptake, while sarcomas frequently demonstrate heterogeneous enhancement due to necrosis, hemorrhage, and irregular vascularity.^21^ Moreover, PET-CT can provide metabolic clues; sarcomas generally exhibit increased FDG uptake owing to their aggressive metabolic activity, unlike myxomas which are metabolically inactive. Hence, employing a multimodal imaging approach—including echocardiography, MRI, and PET-CT—plays a critical role in differentiating malignant from benign cardiac tumors, enabling appropriate surgical planning and early oncologic referral. Treatment strategies varied and included surgical resection alone or in combination with chemotherapy and radiotherapy. Outcomes were heterogeneous, ranging from early recurrence within 6 months to disease-free survival at 26 months, reflecting the aggressiveness and unpredictable course of cardiac UPS. This comparative analysis highlights the critical importance of complete surgical excision, early initiation of multimodal adjuvant therapy, and diligent oncologic follow-up.

We reviewed 18 recently published reports of cardiac undifferentiated pleomorphic sarcoma (UPS), with emphasis on left atrial location, imaging findings, management, and outcomes (Table 4) These cases illustrate the recurring diagnostic pitfalls, particularly the frequent misinterpretation of imaging as benign lesions (eg, myxomas or fibromas), and the subsequent histological confirmation of high-grade UPS after resection. Common patterns include low or variable FDG uptake on PET, and non-specific or fibroma-like appearances on cardiac MRI, underscoring the limitations of imaging alone for definitive diagnosis. Survival outcomes varied widely, with several reports highlighting early recurrence or metastasis despite surgical resection.^21-38^

**Table 4.** Summary of Select Published Cases of Cardiac Undifferentiated Pleomorphic Sarcoma (UPS) Involving the Left Atrium.

Author (year)	Site of tumor	Size (cm)	Imaging features	Treatment	Outcome/follow-up
Yager et al. (2024)	Left atrium (multiple)	3-5	Echo mass, FDG PET low uptake	Resection + bowel metastasis resection	>2-year survival
Gandhi et al. (2024)	Left atrium	4.5	Tumor blush on angiography	Surgical excision	Recurrence at 6 mo
Ha et al. (2023)	Left atrium	4.0	CMR: fibroma-like	Surgical resection	Died 10 mo
Zhang et al. (2024)	Left atrium, pregnancy	5.0	Echo mass, PET low uptake	C-section + resection	Died 8 mo
Yoshida et al. (2023)	RVOT	3.2	RVOT obstruction	Surgical resection	Alive 12 mo
Gishto et al. (2024)	Right atrium	6.0	Echo: irregular mass	Resection	Recurrence 6 mo
Mohammadi et al. (2024)	Left atrium	5.5	PET/CT variable uptake	Surgical resection	Died 9 mo
Wang et al. (2020)	Left atrium	4.0	PET: low uptake (myxoma mimic)	Surgical excision	Alive 6 mo
Seçinti et al. (2022)	Left atrium	3.0	Mimicking myxoma	Resection	Recurrence 1 y
Killian et al. (2023)	Mixed series (atria, ventricles)	Variable	CMR + PET	Resection ± chemo	Median survival < 1 y
Ugarte et al. (2021)	Left atrium	4.8	Echo mass	Surgical excision	Died 14 mo
Alizadehasl et al. (2024)	Left atrium	6.5	LCx encasement	Surgical excision	Alive 10 mo
Zhang et al. (2025)	Left atrium	4.2	CMR mass	Resection	Alive 8 mo
Nguyen et al. (2023)	Left atrium	4.0	Mimicked benign	Resection	Died 7 mo
Prakash et al. (2022)	Right atrium	6.0	Tamponade presentation	Resection	Died 5 mo
Yakovlev et al. (2021)	Right atrium	5.2	Tamponade	Resection	Alive 4 mo
Yates et al. (2025)	Left atrium	4.5	UPS with MMR deficiency	Resection + adjuvant	Alive 1 y
Burkhard-Meier et al. (2025)	Mixed UPS series	—	—	Resection ± chemo/radiation	Median survival 12 mo
Rahbar et al. (2012)	Series (UPS subset)	—	PET differentiation	Surgical excision	PET improved diagnosis
Hu et al. (2024)	Series (UPS subset)	—	PET metabolic parameters	Surgery ± chemo	Outcomes variable

This table compares clinical presentation, imaging findings, histopathology, treatment approaches, and outcomes in 4 reported cases of cardiac UPS. These illustrate variability in imaging appearance, frequent initial misdiagnosis as myxoma, and differing outcomes despite aggressive management strategies.

## Conclusion

This case highlights the diagnostic and therapeutic challenges posed by cardiac undifferentiated pleomorphic sarcoma (UPS), a rare and aggressive malignancy that can closely mimic benign cardiac tumors such as myxomas. Our patient’s initial presentation, combined with suggestive imaging and an early misinterpretation of pathology, underscores the difficulty in establishing a definitive diagnosis prior to surgical excision. The case reinforces the need for heightened clinical suspicion, especially when imaging features are atypical or when there is rapid recurrence after presumed benign tumor resection. Integration of advanced imaging, timely histopathologic reassessment, and multidisciplinary planning is critical to avoid delays in diagnosis and treatment. Furthermore, this case illustrates the importance of structured postoperative surveillance, including multimodal imaging and timely oncology referral. The use of systemic chemotherapy achieved disease stabilization, and close follow-up allowed for monitoring of both tumor activity and cardiac function. In clinical practice, such cases emphasize the need for early referral to specialized cardio-oncology teams, consideration of molecular profiling, and tailored adjuvant strategies to improve outcomes in patients with primary cardiac sarcomas. This report adds to the limited literature guiding clinicians on the optimal pathway for diagnosis, surgical planning, and follow-up in these rare but devastating tumors.

## Take-Home Message

UPS in the heart represents a significant clinical challenge due to its rarity, aggressive behavior, and potential for misdiagnosis are high. The existing literature emphasizes the need for heightened awareness among healthcare professionals regarding this condition. Additionally, there is an urgent requirement for more comprehensive studies to develop standardized treatment protocols and improve patient outcomes. Future research should focus on enhancing early detection methods and exploring targeted therapies to potentially improve survival rates and quality of life for affected patients.

## References

[1] Ramlawi B Leja MJ Abu Saleh WK , et al. Surgical treatment of primary cardiac sarcomas: review of a single-institution experience. Ann Thorac Surg. 2016;101(2):698-702. 26476808 10.1016/j.athoracsur.2015.07.087

[2] Neuville A Collin F Bruneval P , et al. Intimal sarcoma is the most frequent primary cardiac sarcoma: clinicopathologic and molecular retrospective analysis of 100 primary cardiac sarcomas. Am J Surg Pathol. 2014;38(4):461-469. 24625414 10.1097/PAS.0000000000000184

[3] Yusuf SW Bathina JD Qureshi S , et al. Cardiac tumors in a tertiary care cancer hospital: clinical features, echocardiographic findings, treatment and outcomes. Heart Int. 2012;7(1):e4. 10.4081/hi.2012.e4 PMC3366300 22690297

[4] Randhawa JS Budd GT Randhawa M , et al. Primary cardiac sarcoma: 25-year Cleveland clinic experience. Am J Clin Oncol. 2016;39(6):593-599. 25036471 10.1097/COC.0000000000000106

[5] Suh SH Park TH Yoo JN , et al. Primary undifferentiated pleomorphic sarcoma of the left atrium that presented as acute pulmonary edema. Yonsei Med J. 2007;48(1):131-134. 17326257 10.3349/ymj.2007.48.1.131 PMC2627998

[6] Llombart-Cussac A Pivot X Contesso G , et al. Adjuvant chemotherapy for primary cardiac sarcomas: the IGR experience. Br J Cancer. 1998;78(12):1624-1628. 9862574 10.1038/bjc.1998.733 PMC2063231

[7] Look Hong NJ Pandalai PK Hornick JL , et al. Cardiac angiosarcoma management and outcomes: 20-year single-institution experience. Ann Surg Oncol. 2012;19(8):2707-2715. 22476752 10.1245/s10434-012-2334-2

[8] Burke A Virmani R. Tumors of the Heart and Great Vessels. Atlas of Tumor Pathology. 3rd series, fascicle 16 Armed Forces Institute of Pathology; 1996.

[9] Orlandi A Ferlosio A Roselli M , et al. Cardiac sarcomas: an update. J Thorac Oncol. 2010;5(9):1483-1489. 20651614 10.1097/JTO.0b013e3181e59a91

[10] Centofanti P Di Rosa E Deorsola L , et al. Primary cardiac tumors: early and late results of surgical treatment in 91 patients. Ann Thorac Surg. 1999;68(4):1236-1241. 10543485 10.1016/s0003-4975(99)00700-6

[11] Simpson L Kumar SK Okuno SH , et al. Malignant primary cardiac tumors: review of a single institution experience. Cancer. 2008;112(11):2440-2446. 18428209 10.1002/cncr.23459

[12] Abu Saleh WK Ramlawi B Shapira OM , et al. Improved outcomes with the evolution of a neoadjuvant chemotherapy approach to right heart sarcoma. Ann Thorac Surg. 2017;104(1):90-96. 28189277 10.1016/j.athoracsur.2016.10.054

[13] Oliveira GH Al-Kindi SG Hoimes C Park SJ. Characteristics and survival of malignant cardiac tumors: a 40-year analysis of >500 patients. Circulation. 2015;132(25):2395-2402. 26467256 10.1161/CIRCULATIONAHA.115.016418

[14] Donsbeck AV Ranchere D Coindre JM Le Gall F Cordier JF Loire R. Primary cardiac sarcomas: an immunohistochemical and grading study with long-term follow-up of 24 cases. Histopathology. 1999;34(4):295-304. 10231396 10.1046/j.1365-2559.1999.00636.x

[15] Isambert N Ray-Coquard I Italiano A , et al. Primary cardiac sarcomas: a retrospective study of the French sarcoma group. Eur J Cancer. 2014;50(1):128-136. 24135684 10.1016/j.ejca.2013.09.012

[16] Espinoza-Cobos JC Ortiz-Obregón S Blanco-Lemus E Gutiérrez-Tovar G Santos-Martínez LE Moreno-Ruiz LA. High-grade undifferentiated pleomorphic cardiac sarcoma: a rare malignant primary cardiac tumor mimicking double mitral lesion. Arch Cardiol Mex. 2021;91(3):388-390. 34310585 10.24875/ACM.20000320 PMC8351648

[17] Furukawa N Gummert J Börgermann J. Complete resection of undifferentiated cardiac sarcoma and reconstruction of the atria and the superior vena cava: case report. J Cardiothorac Surg. 2012;7:96. 23013671 10.1186/1749-8090-7-96 PMC3514252

[18] Motwani M Kidambi A Herzog BA Uddin A Greenwood JP Plein S. MR imaging of cardiac tumors and masses: a review of methods and clinical applications. Radiology. 2013;268(1):26-43. 23793590 10.1148/radiol.13121239

[19] Rahouma M Arisha MJ Elmously A , et al. Cardiac tumors prevalence and mortality: a systematic review and meta-analysis. Int J Surg. 2020;76:178-189. 32169566 10.1016/j.ijsu.2020.02.039

[20] Rahbar K Seifarth H Schäfers M , et al. Differentiation of malignant and benign cardiac tumors using 18F-FDG PET/CT. J Nucl Med. 2012;53(6):856-863. 22577239 10.2967/jnumed.111.095364

[21] Yager K Fan J Sanford CB Pourfarrokh N Nguyen V. Among the masses: multiple left atrial undifferentiated pleomorphic sarcomas. Ochsner J. 2024;24(4):279-283. 39720829 10.31486/toj.23.0143 PMC11666110

[22] Gandhi R King S Shi B Sasse A. Undifferentiated pleomorphic cardiac sarcoma: a rare and often fatal diagnosis. JACC Case Rep. 2024;29(24):102844. 39822632 10.1016/j.jaccas.2024.102844 PMC11733989

[23] Ha FJ Dimitriou J Huang AL. A rare case of left atrial undifferentiated pleomorphic sarcoma. Eur Heart J Case Rep. 2023;7(10):ytad481. 10.1093/ehjcr/ytad481 PMC10572086 37841043

[24] Zhang J Lin X Yang Y. A case of cardiac undifferentiated pleomorphic sarcoma in late pregnancy: a case report. Sage Open Med Case Rep. 2024;12:2050313X241242894. 10.1177/2050313X241242894 PMC11060023 38689647

[25] Yoshida M Seo M Watanabe T , et al. A case of cardiac undifferentiated pleomorphic sarcoma with right ventricular outflow tract obstruction. Int Heart J. 2023;64(4):779-782. 37518358 10.1536/ihj.23-037

[26] Gishto T Simoni L Kacani A Methoxha S Mehmeti A. A rare case of undifferentiated pleomorphic cardiac sarcoma. Cureus. 2024;16(4):e59183. 10.7759/cureus.59183 PMC11130600 38807801

[27] Mohammadi A Mohammadi M Pazoki M , et al. Clinical presentation, diagnostic evaluation, and management of undifferentiated/unclassified cardiac sarcoma: a case report and literature review. Radiol Case Rep. 2024;19(3):1200-1207. 38259715 10.1016/j.radcr.2023.11.065 PMC10801150

[28] Wang R Shen G Tian R. Primary atrial undifferentiated sarcoma found on FDG PET/CT mimicking cardiac myxoma. Clin Nucl Med. 2020;45(10):833-835. 32804771 10.1097/RLU.0000000000003236

[29] Seçinti İE Gürsoy D Beyaz MO Fansa İ . Undifferentiated pleomorphic sarcoma with focal myogenic differentiation mimicking left atrial myxoma. J Surg Med. 2022;6(4):516-518.

[30] Killian M Barry T Larsen C Alsidawi S. Case series: cardiac sarcoma. Eur Heart J Case Rep. 2023;7(12):ytad546. 10.1093/ehjcr/ytad546 PMC10691649 38046649

[31] Moeri-Schimmel R Pras E Desar I Krol S Braam P. Primary sarcoma of the heart: case report and literature review. J Cardiothorac Surg. 2020;15:104. 32430055 10.1186/s13019-020-01157-4 PMC7236931

[32] Alizadehasl A Hakimian H Dokhani N Pouraliakbar H Firoozbakhsh P . Early presentation of undifferentiated pleomorphic cardiac sarcoma. Radiol Case Rep. 2024;19(10):4308-4311. 39161570 10.1016/j.radcr.2024.06.088 PMC11331714

[33] Zhang J . Diagnosis and Treatment of Primary Cardiac Pleomorphic Undifferentiated Sarcoma: a c ase report. Jour of Clin Cas Rep, Med Imag and Heal Sci. 2025;10(3):1-3.

[34] Prakash S Luis Rayas J Rojas Murguia A , et al. Undifferentiated pleomorphic sarcoma presenting with cardiac tamponade: a case report and review. J Investig Med High Impact Case Rep. 2022;10:23247096221141190. 10.1177/23247096221141190 PMC9720795 36458807

[35] Yakovlev S Kalinskaya A . (2021). A case of undifferentiated pleomorphic cardiac sarcoma. Hear Vessel Transplant. 2021;5:144-149.

[36] Yates CE Connors GW Gurung A Iyer A Wang WYS . Undifferentiated pleomorphic sarcoma with mismatch repair deficiency arising from the heart. JACC Case Rep. 2025;30(13):103532. 40480761 10.1016/j.jaccas.2025.103532 PMC12235140

[37] Burkhard-Meier A Di Gioia D Jurinovic V , et al. Primary cardiac sarcoma: Insights from two decades of multimodal management at LMU Munich. Cardiooncology. 2025;11(1):58. 40571919 10.1186/s40959-025-00359-w PMC12199525

[38] Hu X Yang P Pan D Wang P. 18F-FDG PET/CT metabolic parameters can semi-quantitatively evaluate the nature of the heart and pericardial masses: a retrospective study. Sci Rep. 2024;14(1):16316. 39009884 10.1038/s41598-024-67336-8 PMC11251084

